# Clinical simulation strategies for knowledge integration relating to initial critical recognition and management of COVID-19 for use within continuing education and health-related academia in Brazil: a descriptive study

**DOI:** 10.1590/1516-3180.2020.0155.R2.15062020

**Published:** 2020-08-14

**Authors:** Carolina Felipe Soares Brandão, Gabriela Furst Vaccarezza, João Carlos da Silva Bizario, Aécio Flavio Teixeira de Gois

**Affiliations:** I BSc, MSc, PhD. Professor, Simulation Laboratory of the Medicine Program, Universidade Municipal de São Caetano do Sul (USCS), São Caetano do Sul (SP), Brazil.; II DDS, MSc. Professor of Medicine Program, Universidade Municipal de São Caetano do Sul (USCS), São Caetano do Sul (SP), Brazil.; III MD, MSc, PhD. Director of Medicine Program, Universidade Municipal de São Caetano do Sul (USCS), São Caetano do Sul (SP), Brazil.; IV MD, MSc, PhD. Cardiologist and Adjunct Professor, Disciplines of Emergency and Evidence-Based Medicine, Escola Paulista de Medicina (EPM), Universidade Federal de São Paulo (UNIFESP), São Paulo (SP), Brazil.

**Keywords:** Simulation training, Continuing medical education, Primary health care, Coronavirus, SARS virus, Simulation, SARS-CoV-2, Patient safety

## Abstract

**BACKGROUND:**

The COVID-19 pandemic has led to an immense need to develop training on case recognition and management, with a focus on patients’ and health professionals’ safety at several levels of healthcare settings in Brazil. Different simulation strategies can be included in the diverse clinical care phases for these patients.

**OBJECTIVE:**

To suggest a complete simulation-based training program for Brazilian hospitals and/or academic institutions at this moment of the pandemic.

**DESIGN AND SETTING:**

Descriptive analysis on possible simulated clinical cases using different methodologies, thereby supporting suspected or confirmed COVID-19 patients.

**METHODS:**

This was a reflective theoretical descriptive study on an educational program based on clinical simulation, with four practical phases at different performance and complexity levels. Wearing, handling and adequately disposing of personal protective equipment, along with specific respiratory procedures in different healthcare settings up to intensive care for seriously infected patients were addressed.

**RESULTS:**

This program was designed for application at different Brazilian healthcare levels through different clinical simulation strategies. Summaries of expected performance were suggested in order to standardize technical capacity within these simulation settings, so as to serve these levels.

**CONCLUSIONS:**

Developing training programs for situations such as the current COVID-19 pandemic promotes safety not only for patients but also for healthcare workers. In the present context, clear definition of which patients need hospital outpatient or inpatient care will avoid collapse of the Brazilian healthcare system. Institutions that do not have simulated environments can, through the examples described, adopt procedures to promote didactic information in order to help healthcare professionals during this time.

## INTRODUCTION

The current global pandemic of coronavirus disease (COVID-19) has forced medical trainees throughout the world to suspend their clinical training and transition largely to online lectures. Through removing trainees from clinical settings, medical program managers hope to reduce the risk of person-to-person spread of infection.^1^

Clinical simulation has the objective of replicating scenarios that are as close as possible to reality, in order to train healthcare professionals in diverse clinical situations that demand clinical thinking, as well as simultaneous attitudinal and procedural abilities.^2^

Simulation-based medical education (SBME) is a tool within medical training that has quickly expanded in use over recent years and has enabled great advances. In particular, it has been lauded for its improvement of medical education, coupled with cost savings and protection of patients.^3^

Diverse simulated strategies can be used, depending on the expertise level of participants and the central objective at hand, as well as the availability of resources and specialists in the method and topic to be developed.^2^

Situations requiring extreme care, rare clinical cases and situations requiring emergency training are benefited through simulation strategies. These strategies take into consideration the safety of the patients and health professionals involved, within a controlled environment, with repetition and good rates of knowledge absorption, compared with passive learning methods.^2^

COVID-19 belongs to the beta-coronavirus family. These are ribonucleic acid (RNA) viruses with high mutation rates enabling adaptation to diverse host organisms, and with fast dissemination among humans. Human-to-human transmission of COVID-19 occurs through respiratory droplets and fomites. There is also the possibility of transmission during the disease incubation period or by patients with only mild symptoms, but this remains poorly understood.^4^

The clinical presentation is similar to that of the common flu, with nonspecific symptoms that can include pyrexia (fever), indisposition, coughing, pharyngitis (sore throat) and rhinorrhea (runny nose), among others. In the majority of cases (around 80%), no special treatment will be needed. However, one in every six people who acquire COVID-19 may present the severe form of the illness, with acute airway impairment.^4^

In the current scenario, which has been declared to be a pandemic, it is mandatory that healthcare professionals are trained to deal with COVID-19 at the different levels of the healthcare system, with the aim of optimizing resources and promoting safe, high-quality care. Cases need to be registered through an electronic form within the first 24 hours from the start of clinical suspicion. Currently, there are many cases of community transmission in Brazil and it is essential that all teams are trained in order not to burden and overload the healthcare services unnecessarily, including bed and supply use, which could culminate in a chaotic healthcare situation.^5^

## OBJECTIVE

To describe different clinical simulation strategies, in which the aim is to train healthcare professionals to recognize and manage COVID-19 within the Brazilian healthcare system.

## METHODS

This was a reflective theoretical descriptive study on an educational program based on clinical simulation, with four practical phases at different levels of performance and complexity.

This study addressed the use of a variety of different procedures. Wearing, handling and adequately disposing of personal protective equipment (PPE) was assessed, including use of gloves, medical masks, goggles or face shields, and gowns. Equipment for specific procedures such as respirators (i.e. N95 or FFP2 standard or equivalent) and aprons was also addressed, in different healthcare settings up to intensive care for seriously infected patients.

## RESULTS

This program was designed to be applied at different levels of Brazilian healthcare through different clinical simulation strategies. Summaries of expected performance were suggested in order to standardize technical capacity within these simulation settings, thus making it possible to serve the different levels of care within healthcare provision in Brazil.

### Clinical simulation strategies

In starting to train the healthcare team, we considered that it was essential to standardize concepts and practice procedures using low-fidelity manikins (task trainers). In addition, there was a need for reinforcement of the following procedures: basic hand hygiene, adequate use of individual protection equipment, the notification system for suspicious cases, use of peripheral and central accesses, intraosseous puncture, orotracheal intubation, collection of secretions, use of swabs, team movement and putting patients into the prone position. These were deemed to be priorities for trained, with direct feedback.

Phase 1 considered standardized patients, and simulation was used in accordance with the local possibilities in the primary healthcare system, with a focus on anamnesis and physical examination for decision-making to recognize the disease and provide initial management. Phase 2 considered standardized patients, and simulation was used in accordance with the local possibilities in the primary or secondary healthcare system, with a focus on anamnesis and physical examination for decision-making, to recognize the disease and provide initial management. In this context, debriefing so that the participants could reflect and learn was mandatory. The rapid cycle of deliberate practices suggested in phase 3 of the training comprised simulations that were interrupted so that immediate feedback could be provided to the facilitator, with the aims of performance enhancement and allowing many repetitions.^6^ In this phase, it was suggested that patients in need of hospitalization should be taken care of. Lastly, to end the training, phase 4 consisted of a classic simulation followed by debriefing, which concentrated on taking care of critical patients with deterioration of the hemodynamic situation, as presented in Table 1.

**Table 1.** Description of methodologies and content to be addressed during COVID-19 training

Methodology	Context	Objectives	Topics to be discussed
Phase 1: Task trainer	Specific-ability manikins for handwashing, orotracheal intubation, peripheral and central access and intraosseous puncture	Fundamental procedural review from initial to critical care management	PPEs, indications, counterindications, good practice review, COVID-19 patient intubation care and the risks of noninvasive ventilation during transmission
Phase 2: Standardized patient	To be cared for within the primary or secondary healthcare system	Triage; initial recognition and management; and knowing the parameters and actions for patients who do not need hospitalization	General parameters, anamnesis and physical examination
Phase 3: Rapid-cycle deliberate practice	Simulator for cases of medium or high levels of complexity, for initial management in an emergency care unit	Recognition and management of patients who need hospitalization; symptomatic treatment and discussion of complementary examinations	Reassessment; discussion of ventilation for adequate support; making a prognosis; and transportation to critical care unit
Phase 4: Standard simulation	Critical care patients in an intensive care unit	Critical care; circulatory repercussions; and specific actions	Discernment of differences in relation to acute respiratory distress syndrome (ARDS); avoidance of excess volume and maintenance of negative balance

PPE = personal protective equipment.

**Figure 1.**
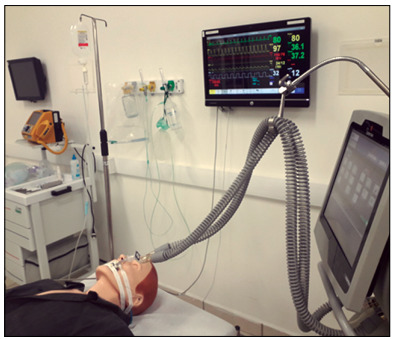
Example of simulation in the emergency room. Orotracheal intubation within the specifications for COVID-19 was performed.

**Figure 2.**
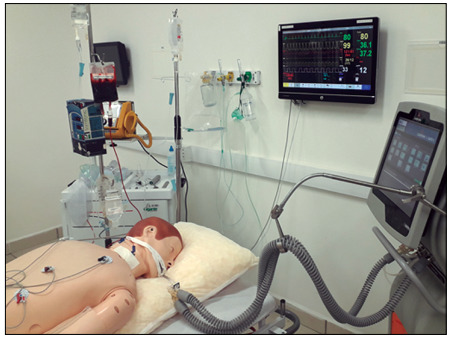
Example of standard simulation. Critical patient with circulatory impairment in prone position.

*In situ* simulation, which occurs inside the real care environment, is advisable when possible. However, the logistics for this are much more complex logistics and this can be more taxing, since the resources used in this care service are the same as what is available for real patients. In this article, the use of specific training spaces is described, in academic and hospital settings (Figures 1 and 2).

### Scenario flowchart

The main objective of this study was to create scenarios that would prepare professionals to determine which patients present symptoms that need hospitalization, and which patients only require examinations and guidance, with reassessment if necessary.

#### Phase 1

Clinical case: Patient K.G.R., 43-year-old female, with fever for one day, dry cough and headache. She goes to a primary care unit for treatment.

- She says that she does not have any allergies or any routine use of medications.
- She says that she has not had any surgery or recent previous pathological conditions.
- Normal water and food intake.
- She has not traveled recently and has not been exposed to any international travelers.

Key points: Presence of fever is confirmed; anamnesis and detailed physical examination are performed; previous records and current use of medication are investigated.

Actions: Pertinent medication is prescribed; patient is advised to stay at home; no examinations using nasal and oropharyngeal swabs are requested.

Simulation: A standardized patient or a simulator that is available can be used; primary care material practice.

Debriefing: Discussion should be focused on parameters that are not criteria for hospitalization and examination requests. Communication abilities are needed for effective and clarifying orientation for patients, who will be anxious and afraid of having COVID-19.

Consider: Primary healthcare is the first point of contact with the healthcare system, for individuals, families and communities. In relation to managing COVID-19, primary healthcare needs to take on a proactive role as the care flow coordinator, i.e. to organize the actions, sequence the flow of care and its continuation, with emphasis on family and community orientation.

#### Phase 2

**Figure 3.**
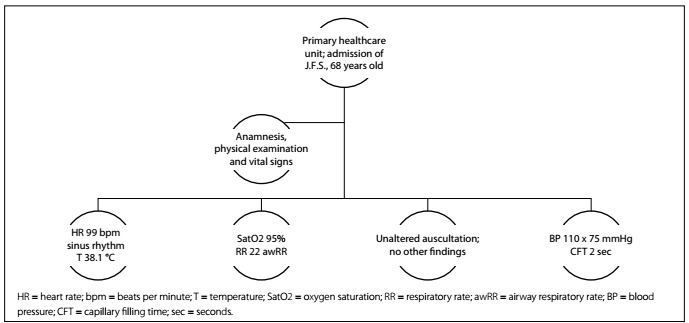
Initial scenario pattern in primary healthcare for Phase 2.

**Figure 4.**
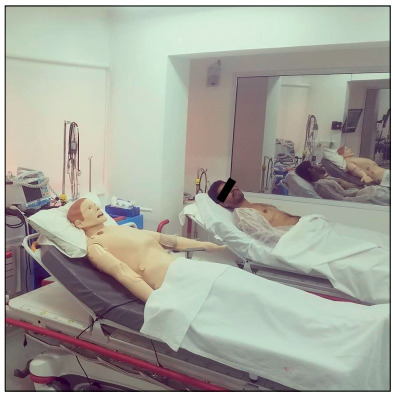
Example of standardized patient (actor) or simulator as an option in scenarios of Phases 1 and 2.

Clinical case: Patient J.F.S., 63-year-old male, with fever for one day, dry cough and coryza. He goes to a primary care unit for treatment (Figures 3 and 4).

- He says that he does not have any allergies, and is using beta-blockers and statins.
- He says that he has not had any surgery or recent previous pathological conditions.
- Normal water and food intake.
- He has not traveled recently and has not been exposed to any international travelers.

Key points: Presence of fever is confirmed; anamnesis and detailed physical examination are performed; previous records and current use of medication are investigated.

Actions: Pertinent medication is prescribed; patient is advised to stay at home; no examinations using nasal and oropharyngeal swabs are requested. Prescription of oseltamivir (75 mg twice a day) can be considered for this at-risk group. Reassessment orientation can be provided if necessary.

Simulation: A standardized patient or a simulator that is available can be used; primary care material practice.

Debriefing: Discussion should be focused on parameters that are not criteria for hospitalization and examination requests. Communication abilities are needed for effective and clarifying orientation for patients, who will be anxious and afraid of having COVID-19.

Consider: Classifying the severity of the patient’s flu-like syndrome will define whether the patient will be kept within primary care or will be referred to specialist centers, emergency centers or hospitals.

Patients presenting less than 95% oxygen saturation in room air, signs of respiratory discomfort, hypotension or acute respiratory insufficiency need to be sent for specialized attention.^1^

#### Phase 3

**Figure 5.**
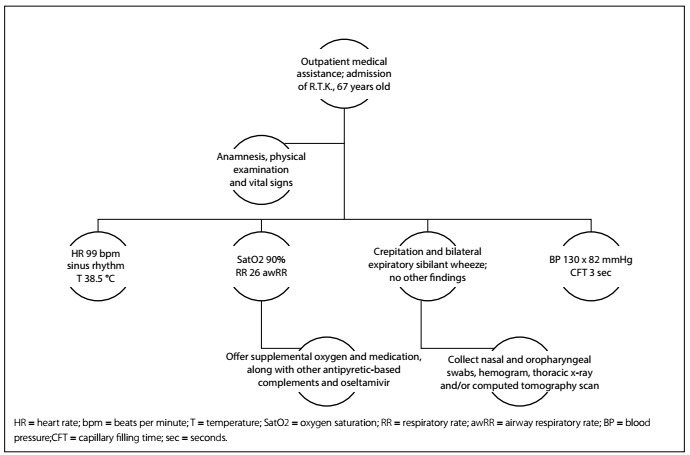
Scenario pattern indicative of investigation and hospitalization.

Clinical case: R.T.K., 67-year-old female with fever for two days, body pain, sore throat and dry cough. She seeks medical care and treatment at a hospital outpatient clinic (Figure 5).

- She says that she does not have any allergies, and is using metformin hydrochloride.
- She says that he has not had any recent surgery or recent previous pathological conditions.
- Diminished water and food intake.
- She has not traveled recently and has not been exposed to any international travelers.

Key points: Need for hospitalization is identified through general assessment of the patient; need to understand which patients are at high risk; directed requests for complementary exams need to be made. Use of complementary oxygen and notification needs to be discussed.

Simulation: A medium to high-fidelity simulator in which parameters can be seen as pertinent alterations (auscultation) should be used. A medical assistance room with supplemental oxygen material is required.

Rapid-cycle deliberate practice: The specialist will give immediate feedback regarding the participants’ behavior. The discussion should focus on the parameters that are criteria for hospitalization and on understanding which specific examinations that can be requested will have prognostic value.

#### Phase 4

This is the same patient as in Phase 3, but with worsening of the patient’s condition.

**Figure 6.**
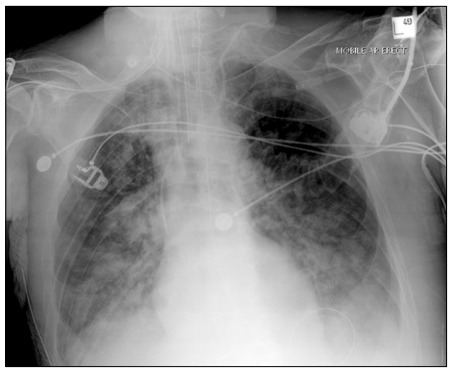
Chest X-ray used for COVID-19 training. Case courtesy of Prof Frank Gaillard, Radiopaedia.org.

**Figure 7.**
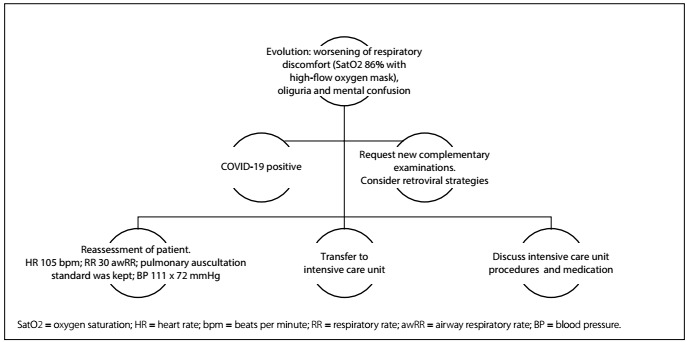
Initial pattern of patient in critical care.

Clinical case: R.T.K. 67-year-old female with fever for two days, body pain, sore throat and dry cough. She sought medical care and treatment at a hospital outpatient clinic and was admitted to that hospital. Examinations were requested (Figures 6 and 7).

Key points: Her condition has worsened, with the need to ensure airway patency. Complementary examinations that can be used include: glutamic oxaloacetic transaminase (GOT), glutamic pyruvate transaminase (GPT), D-dimer, troponin, arterial blood gas test, urea, creatinine, sodium (Na), potassium (K), lactate dehydrogenase, C-reactive protein (CRP) and ferritin.

Simulation: A medium-fidelity or high-fidelity simulator in which parameters can be seen as pertinent alterations should be used. Laboratory tests and imaging examinations need to be made available at this stage of care, to aid in decision-making. A medical assistance room equipped with emergency care is needed.

Debriefing: The focus should be on actions to be taken before clinical deterioration, airway handling and mechanical ventilation.

Consider: The guidelines for the novel coronavirus are frequently changing, as more information about the virus is received. Hospital infection control committee recommendations regarding on prevention also vary among institutions. The most up-to-date guidelines need to be reviewed and discussed with the committee’s team before executing any simulation.

Lastly, use of an tool within the checklist format to summarize the expected performance at each phase is suggested. It should be ensured that discussions become homogeneous within all groups that are trained (Table 2).

**Table 2.** Performance tools expected to be used within each training phase

Scenarios	Checklist
Phase 1	Patient identification Use of relevant personal protective equipment Directed anamnesis Physical examination with precautions for COVID-19 Doctor-patient communication Clinical reasoning
Phase 2	Patient identification Use of relevant personal protective equipment Directed anamnesis Physical examination with precautions for COVID-19 Doctor-patient communication Clinical reasoning Requesting and carrying out complementary examinations
Phase 3	Patient identification Use of relevant personal protective equipment Directed anamnesis Physical examination with precautions for COVID-19 Doctor-patient communication Clinical reasoning Requesting and carrying out complementary examinations Discussion of hospitalization with the patient
Phase 4:	Patient identification Use of relevant personal protective equipment Directed anamnesis Physical examination with precautions for COVID-19 Doctor-patient communication Clinical reasoning Requesting and carrying out complementary examinations Emergency airway procedure Referral to intensive care unit

## DISCUSSION

Within healthcare education, patient-focused learning is fundamental. It is more meaningful and more motivating than any other educational strategy.

However, safety-related issues, ethics and the need for efficient learning within a short time make clinical simulation an option that promotes contextualization, motivation, learning feedback and, especially, concrete reproduction of the applicability of the acquired knowledge. This is one of the bases of andragogy, i.e. adult education.^2^

In a recent study, a simulation mannequin was used to analyze the effectiveness of personal protective equipment (PPE) during the COVID-19 outbreak and to subsequently adjust PPE standards accordingly.^7^ Simulation can be used to address issues ranging from proper donning of PPE to patient management. It allows trainees to adhere to the bans on public gatherings that are being enforced by local governments and thus enables them to continue their medical education. This can be accomplished through eliminating human contact at simulation centers, remotely distributing virtual reality technology that is currently used within anatomy education, and/or integrating virtual simulation programs into online curricula.^7^

Care provision during the COVID-19 pandemic still presents uncertainties and difficulties regarding triage, initial care and critical patient management. This justifies implementation of training based on the most relevant information on how to manage these patients. The pedagogical structure suggested here can also be used for several other situations, for training through clinical simulation.

However, one limitation of the present study relates to the dynamic course of the COVID-19 pandemic and the few education-focused published papers addressing this. Therefore, the scenarios described here may need adaptations to align them at all levels of care as new research on the conduct and protocols to be followed moves forward.

## CONCLUSIONS

Use of different clinical simulation strategies can contribute effectively to healthcare professionals’ training at all levels of the Brazilian healthcare system. It is suggested that educational institutions should help their care partners with potential specific training as much as possible. Developing training programs in situations resembling the current COVID-19 pandemic promotes safety not only for patients but also for the professionals involved. Within the current context, determining which patients need hospital assistance or need to be hospitalized will avoid collapse of care provision. Institutions that do not have simulation environments can, through the examples described here, adopt other ways of promoting didactic information, in order to help healthcare professionals during this difficult time.
